# Shrinking Lung Syndrome in Primary Sjögren Syndrome

**DOI:** 10.4274/balkanmedj.galenos.2020.2020.4.143

**Published:** 2020-10-23

**Authors:** Sadettin Uslu, Aydan Köken Avşar, Yeşim Erez, İsmail Sarı

**Affiliations:** 1 Clinic of Rheumatology, Ömer Halisdemir University Bor Physical Medicine and Rehabilitation Training and Research Hospital, Niğde, Turkey; 2 Department of Rheumatology, Dokuz Eylül University School of Medicine, İzmir, Turkey

A 48-year-old woman presented with a three months history of dyspnea, fever, and pain and swelling in her hand and wrist joints had an unremarkable past medical history. Detailed clinical history revealed she had dryness of the eyes and mouth for three years. On examination, she had a fever of 38°C, tachypnea (18/minute), reduced breath sounds in the left lower lung zone, arthritis in her wrists, metacarpophalangeal, and proximal interphalangeal joints. Erythrocyte sedimentation rate (68; range, 0-20 mm/hr) and C-reactive protein (108; range, 0-5 mg/L) were increased in the laboratory. Antinuclear antibody testing was positive in high titers (1/1600, granular staining) along with a positive anti-Ro/SS-A. The rest of the tests carried out in the laboratory, including urine analysis, anti-ds-DNA, anti-Sm, and complement were normal. Chest X-ray revealed an elevated hemidiaphragm and loss of volume on the left lung with normal parenchyma (Figure 1A). Computed tomography (CT) showed thinning of the diaphragmatic crura (Figure 1B). Fluoroscopy revealed diaphragmatic dysfunction on the left side (Figure 2). Pulmonary function tests were consistent with a restrictive pattern (FVC: 65%) and carbon monoxide diffusion capacity (DLCO: 57%) was reduced. Schirmer’s test was positive (<5 mm in 5 minutes for both eyes). Further minor salivary gland biopsy was showing diffuse lymphocytic infiltrations with a focus score of 4. The patient was diagnosed as primary Sjögren’s syndrome (pSS) and shrinking lung syndrome (SLS), and put on azathioprine (AZA; 2 mg/kg/day) and prednisone (0.5 mg/kg/day). Significant clinical improvement was observed on her dyspnea and arthritis within few days of the treatment and complete radiologic improvement achieved in a month (Figure 1C, D). The study was approved by the local ethics committee (IProtocol number: 127535) and a written informed consent was obtained from the patient.

SLS is a rare pulmonary manifestation of inflammatory rheumatic diseases, which is mostly reported in systemic lupus erythematosus. The main findings of SLS includes; dyspnea, pleuritic chest pain, progressive decrease in lung volumes, the elevation of the diaphragm, and the absence of significant parenchymal and pleural disease on thorax CT. The underlying pathology of SLS is unclear, but researchers suggested the following as possible mechanisms: 1) myopathy or myositis affecting the diaphragm or intercostal muscles, 2) diaphragmatic dysfunction secondary to pleural adhesions, 3) phrenic neuropathy, and 4) pleuritic pain-related diaphragmatic dysfunction through reflex inhibition of diaphragmatic activation (1). Neurological involvement is a well-known complication of pSS, and a wide variety of neurological symptoms could be seen in the course of the disease. A small study performed in pSS patients with neuropathy reported that about 5% of these patients had phrenic nerve palsy (2). Therefore, we may hypothesize that phrenic neuropathy, operating alone or in combination with the others mentioned above, could explain the SLS in the current case. Presently, there is no established treatment for pSS associated SLS but, similar to our report, corticosteroids in combination with immunosuppressives (AZA, cyclophosphamide or rituximab) have been suggested in the case series. In conclusion, SLS should be considered in the differential diagnosis of pSS with dyspnea, or pleuritic chest pain, and patients should be put on immunosuppressants as they provide a rapid and effective response to the condition.

## Figures and Tables

**Figure 1:**
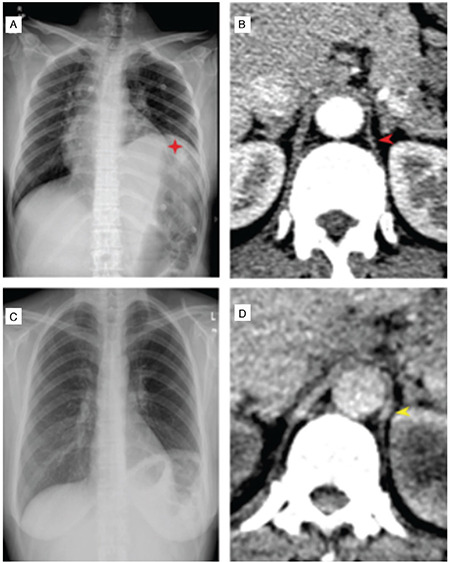
A) Elevated hemidiaphragm and loss of volume on the left lung with normal bilateral lung parenchyma on chest X-ray. B) Thorax axial CT image showing the thinning of the diaphragmatic crura (red arrowhead) at the time of presentation. C) The improvement in the lung volume and diaphragm and D) diaphragmatic crura (yellow arrowhead) after immunosuppressive treatment.

**Figure 2:**
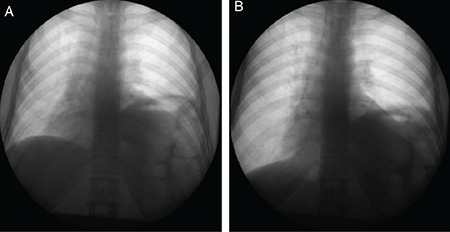
Fluoroscopy imaging of the lung showing reduced left diaphragmatic excursion. Images are obtained in A) Expiration and B) Inspiration.
